# Supplementary material for: Multi‐dataset identification of innovative feature genes and molecular mechanisms in keratoconus
Source: J Cell Mol Med. 2024 Sep 19;28(18):e70079. doi: 10.1111/jcmm.70079 (PMC11412914; doi:10.1111/jcmm.70079)
Supplement: Supplementary file 2 — Material S2. [file JCMM-28-e70079-s002.zip › 7.GSEA analysis/4.MYRF/KEGG_CHRONIC_MYELOID_LEUKEMIA.html]

Details for gene set KEGG\_CHRONIC\_MYELOID\_LEUKEMIA[GSEA]

|  || Dataset | MYRF.group.cls#h\_versus\_l |
| Phenotype | group.cls#h\_versus\_l |
| Upregulated in class | l |
| GeneSet | KEGG\_CHRONIC\_MYELOID\_LEUKEMIA |
| Enrichment Score (ES) | -0.38700062 |
| Normalized Enrichment Score (NES) | -1.5673198 |
| Nominal p-value | 0.06986028 |
| FDR q-value | 0.21043545 |
| FWER p-Value | 0.664 |
Table: GSEA Results Summary

  

Fig 1: Enrichment plot: KEGG\_CHRONIC\_MYELOID\_LEUKEMIA      
 Profile of the Running ES Score & Positions of GeneSet Members on the Rank Ordered List

  

| SYMBOL | TITLE | RANK IN GENE LIST | RANK METRIC SCORE | RUNNING ES | CORE ENRICHMENT || 1 | PIK3R5 | na | 661 | 0.528 | 0.0342 | No |
| 2 | CBLB | na | 3959 | 0.256 | -0.0352 | No |
| 3 | SOS1 | na | 6706 | 0.172 | -0.0970 | No |
| 4 | PIK3R1 | na | 7105 | 0.161 | -0.0923 | No |
| 5 | MECOM | na | 7274 | 0.157 | -0.0813 | No |
| 6 | BRAF | na | 8198 | 0.133 | -0.0945 | No |
| 7 | KRAS | na | 9371 | 0.108 | -0.1174 | No |
| 8 | PIK3CG | na | 9794 | 0.100 | -0.1196 | No |
| 9 | CBL | na | 9933 | 0.097 | -0.1138 | No |
| 10 | AKT2 | na | 10210 | 0.092 | -0.1124 | No |
| 11 | PIK3CA | na | 11255 | 0.074 | -0.1351 | No |
| 12 | E2F3 | na | 11256 | 0.074 | -0.1276 | No |
| 13 | SHC3 | na | 11433 | 0.071 | -0.1255 | No |
| 14 | PTPN11 | na | 11569 | 0.069 | -0.1224 | No |
| 15 | E2F2 | na | 12461 | 0.057 | -0.1424 | No |
| 16 | PIK3R2 | na | 12535 | 0.056 | -0.1388 | No |
| 17 | RB1 | na | 12759 | 0.053 | -0.1399 | No |
| 18 | SOS2 | na | 12783 | 0.053 | -0.1353 | No |
| 19 | TP53 | na | 12977 | 0.051 | -0.1357 | No |
| 20 | IKBKB | na | 12994 | 0.050 | -0.1311 | No |
| 21 | SMAD4 | na | 13356 | 0.045 | -0.1370 | No |
| 22 | MDM2 | na | 13578 | 0.043 | -0.1390 | No |
| 23 | MAP2K2 | na | 14012 | 0.038 | -0.1477 | No |
| 24 | CDKN1B | na | 14078 | 0.037 | -0.1458 | No |
| 25 | CHUK | na | 14348 | 0.034 | -0.1502 | No |
| 26 | MAPK3 | na | 14382 | 0.034 | -0.1477 | No |
| 27 | CBLC | na | 14386 | 0.034 | -0.1444 | No |
| 28 | CTBP2 | na | 14461 | 0.033 | -0.1432 | No |
| 29 | CCND1 | na | 14657 | 0.031 | -0.1457 | No |
| 30 | CTBP1 | na | 14847 | 0.029 | -0.1482 | No |
| 31 | STAT5B | na | 15287 | 0.025 | -0.1584 | No |
| 32 | BCR | na | 15503 | 0.023 | -0.1623 | No |
| 33 | PIK3CB | na | 17199 | 0.007 | -0.2105 | No |
| 34 | SMAD3 | na | 17747 | 0.002 | -0.2261 | No |
| 35 | HDAC2 | na | 17814 | 0.001 | -0.2279 | No |
| 36 | AKT1 | na | 18101 | -0.001 | -0.2360 | No |
| 37 | HDAC1 | na | 19552 | -0.017 | -0.2762 | No |
| 38 | CDKN2A | na | 19779 | -0.019 | -0.2807 | No |
| 39 | MAPK1 | na | 20406 | -0.027 | -0.2961 | No |
| 40 | PIK3CD | na | 20433 | -0.027 | -0.2942 | No |
| 41 | PIK3R3 | na | 20464 | -0.027 | -0.2923 | No |
| 42 | IKBKG | na | 21687 | -0.042 | -0.3234 | No |
| 43 | RAF1 | na | 21720 | -0.042 | -0.3200 | No |
| 44 | E2F1 | na | 21781 | -0.043 | -0.3174 | No |
| 45 | HRAS | na | 21856 | -0.044 | -0.3151 | No |
| 46 | TGFB2 | na | 22177 | -0.048 | -0.3195 | No |
| 47 | BAD | na | 23795 | -0.072 | -0.3589 | No |
| 48 | ABL1 | na | 23912 | -0.074 | -0.3548 | No |
| 49 | GRB2 | na | 24215 | -0.079 | -0.3555 | No |
| 50 | SHC1 | na | 24263 | -0.080 | -0.3488 | No |
| 51 | CDK4 | na | 24674 | -0.088 | -0.3518 | No |
| 52 | TGFBR2 | na | 24993 | -0.093 | -0.3516 | No |
| 53 | CRK | na | 25398 | -0.101 | -0.3531 | No |
| 54 | TGFBR1 | na | 25528 | -0.103 | -0.3464 | No |
| 55 | CDK6 | na | 25789 | -0.108 | -0.3430 | No |
| 56 | STAT5A | na | 25954 | -0.112 | -0.3364 | No |
| 57 | MYC | na | 27707 | -0.153 | -0.3716 | Yes |
| 58 | NFKB1 | na | 28241 | -0.168 | -0.3701 | Yes |
| 59 | BCL2L1 | na | 28417 | -0.173 | -0.3577 | Yes |
| 60 | ARAF | na | 28444 | -0.174 | -0.3409 | Yes |
| 61 | CRKL | na | 29092 | -0.194 | -0.3400 | Yes |
| 62 | NRAS | na | 30019 | -0.224 | -0.3442 | Yes |
| 63 | MAP2K1 | na | 30150 | -0.229 | -0.3248 | Yes |
| 64 | RELA | na | 31135 | -0.271 | -0.3259 | Yes |
| 65 | NFKBIA | na | 31525 | -0.292 | -0.3078 | Yes |
| 66 | GAB2 | na | 31969 | -0.316 | -0.2887 | Yes |
| 67 | RUNX1 | na | 32001 | -0.317 | -0.2576 | Yes |
| 68 | SHC4 | na | 32535 | -0.353 | -0.2374 | Yes |
| 69 | TGFB1 | na | 32868 | -0.377 | -0.2090 | Yes |
| 70 | TGFB3 | na | 33024 | -0.389 | -0.1742 | Yes |
| 71 | CDKN1A | na | 33771 | -0.483 | -0.1471 | Yes |
| 72 | SHC2 | na | 34640 | -0.800 | -0.0914 | Yes |
| 73 | AKT3 | na | 34696 | -0.932 | 0.0009 | Yes |
Table: GSEA details [plain text format]

  

Fig 2: KEGG\_CHRONIC\_MYELOID\_LEUKEMIA      
 Blue-Pink O' Gram in the Space of the Analyzed GeneSet

  

Fig 3: KEGG\_CHRONIC\_MYELOID\_LEUKEMIA: Random ES distribution      
 Gene set null distribution of ES for **KEGG\_CHRONIC\_MYELOID\_LEUKEMIA**
